# Outcome of patients with primary glioblastoma in Chile: single centre series

**DOI:** 10.3332/ecancer.2021.1184

**Published:** 2021-02-10

**Authors:** Mariana Sinning, Michael Frelinghuysen, Marcela Gallegos, Andrés Cordova, Patricio Paredes, Conrado Vogel, Emi Sujima, Carlos Kamiya-Matsuoka, Felipe Valdivia

**Affiliations:** 1 Departamento de Psiquiatría y Neurología, Clínica Alemana Santiago, Av Manquehue Norte 1410, Vitacura, Santiago, Chile; 2 Departamento de Oncología, Clínica Alemana Santiago, Av Manquehue Norte 1410, Vitacura, Santiago, Chile; 3 Servicio Oncología, Hospital Guillermo Grant Benavente, San Martín 1436, Concepción, Chile; 4 Departamento Laboratorio, Banco de Sangre y Anatomía Patológica, Clínica Alemana Santiago, Av Manquehue Norte 1410, Vitacura, Santiago, Chile; 5 Departamento de Imágenes, Clínica Alemana de Santiago, Av Manquehue Norte 1410, Vitacura, Santiago, Chile; 6 Department of Neuro-oncology, The University of Texas MD Anderson Cancer Center, 1515 Holcombe Blvd, Texas, USA; 7 Departamento de Cirugía, Clínica Alemana de Santiago, Av Manquehue Norte 1410, Vitacura, Santiago, Chile; a https://orcid.org/0000-0002-4445-7796

**Keywords:** glioma, glioblastoma, Latin America, Chile

## Abstract

**Background:**

Glioblastoma (GBM) is the most common and most aggressive primary malignant brain tumour. The standard of care is surgical resection, followed by radiotherapy with concurrent and adjuvant temozolomide. In Latin America, there is scarcity of information about the incidence of GBM and even less data regarding outcomes. In this study, we describe the clinicopathologic features, management and outcomes of GBM patients.

**Methods:**

We describe a single-centre multidisciplinary team experience in managing GBM patients over an 11-year period (Jan 2005 to Dec 2016). Pathology was reviewed by the pathology collaborator and retrospective chart review performed for treatment and clinical outcomes.

**Results:**

We identified 74 patients (50 males) with diagnosis of GBM. Median age at diagnosis was 58 years (range 24–79 years), and median Karnofsky performance status was 80%. Forty-three (58.1%) went to gross total resection, 20 (27%) partial resection and 11 (14.9%) biopsy. Sixty-four (87%) patients received Stupp regimen. The median overall survival (OS) was 13.9 months (standard error (SE) 1.71; 95% confidence interval (CI), 10.56–17.23). In patients treated according to Stupp regimen, the progression-free survival (PFS) was 10 months (SE 1.8; 95% CI, 6.481–13.519), the selfcare survival was 11.8 months (SE 1.61; 95% CI, 8.632–14.968) and the OS was 16.1 months (SE 1.53; 95% CI, 13.01–19.099).

**Conclusions:**

This study reports the most complete analysis of epidemiology, clinical management and outcomes of patients with diagnosis of GBM in Chile treated with Stupp regimen. The PFS and OS are consistent with reports of US and Europe.

## Introduction

Glioblastoma (GBM) is the most common glioma in adults and it is still a disease of poor prognosis despite a vast scope of research. The standard of care typically includes maximal safe surgical resection followed by radiotherapy (60 Gy in 30 fractions) with concomitant and adjuvant temozolomide (TMZ). The median survival for most patients from the time of diagnosis ranges from 12.1 to 14.6 months [1].

There is a great variability in central nervous system (CNS) cancer incidence between regions. The highest rates are observed in Europe and range between 8 and 9 per 100,000 population, whereas the lowest rates (mainly in Asia) range between 2.4 and 4 per 100,000 [2]. Similar incidence rates are seen in Latin America (Central and South America). The lowest (Costa Rica) and highest (Brazil) incidences range between 4.5 and 13.3 per 100,000, respectively [2]. Furthermore, a pilot cancer registry study at Chile reported that the age-standardised incidence rate of malignant brain tumour, between 2003 and 2007, was 1.8–4.6 per 100,000 for men and 2–3.6 per 100,000 for women [3]. To date, there is incomplete data of the incidence of CNS tumours by histological diagnosis.

The aim of the current study is to report the incidence, management and outcomes of patients with diagnosis of GBM at the Clínica Alemana at Santiago (CAS), Chile, between 2005 and 2016, the period of time in which Stupp regimen was implemented.

## Material and methods

We conducted a retrospective data analysis of the CAS institutional database of all adults with diagnosis under the International classification of diseases 10^th^ revision (ICD-10) code, C71 for malignant neoplasm of brain. We conducted a search between January 2005 and December 2016 of all electronical medical records. This study was approved by the CAS Institutional Review Board/Institutional Ethics Committee. Demographic, treatment and survival data were collected from the database. We then included: all adult patients (>18 years) with intracranial histologically proved primary GBM and sufficient follow up information about therapy. In all cases, a neuropathologist confirmed the diagnosis using standard diagnostic classification (World Health Organization Classification System, WHO 2007) [4].

The date of death was obtained through the patients’ national death certificate.

The institutional electronic medical records were reviewed in order to obtain epidemiological information, clinical presentation, postoperative Karnofsky performance status (KPS), tumour location, extent of surgery (biopsy, partial resection (PR) or gross total resection (GTR) determined by postoperative magnetic resonance imaging of brain; GTR defined by resection of >90% of the enhancing lesion) and adjuvant treatment. O^6^-methylguanine-DNA methyl-transferase (MGMT) promoter methylation (by methylation-specific polymerase chain reaction (PCR)) and isocitrate dehydrogenase1 (IDH1) and IDH-2 status (by immunohistochemistry and PCR/direct sequencing) have been available since 2015 in our institution and collected for the current study.

All study data were collected and managed using Research Electronic Data Capture (REDCap) tools hosted at CAS [5].

Survival analysis includes progression-free survival (PFS), overall survival (OS) and selfcare survival (ASS). PFS was defined as the duration between the date of initial histologic diagnosis (surgery) to the date of tumour progression (first recurrence), death or last follow-up. Progression was confirmed based on radiographic parameters by Response Assessment in Neuro-Oncology criteria and/or clinical parameters such as neurological decline or death. OS was defined as the duration between the date of initial diagnosis to the date of death or last follow-up. Death was confirmed by review of medical records, death certificate and/or the social security index database.

Selfcare survival (SS) was defined as the time from initial diagnosis (surgery) to the last follow up with KPS of 70% or more; the survival time of the patient being able to perform the basic activities of the daily living.

Cox univariable regression was made for the variables; age at diagnosis, presence of comorbidity, Charlson Comorbidity Index (CCI), presence/absence of GTR, presence/absence of radiotherapy, presence/absence concomitant TMZ, postoperative KPS, presence of epilepsy and reoperation. Those variables with significance (*p* < 0.05) were included for multivariable analysis. Kaplan–Meier curves and Log-rank test were used for survival comparison throughout variables in study. Statistical analysis was done using SPSS (IBM Corp. 2012, Version 22.0. Armonk, NY) and Microsoft Excel 2010.

## Results

**Figure 1.**
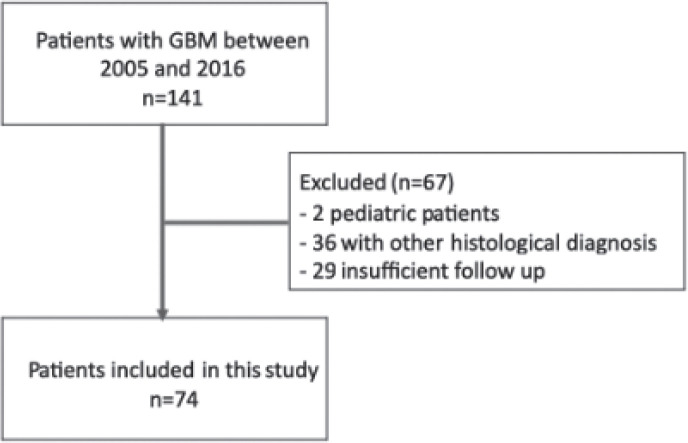
Patients included in the study.

We identified a total of 141 patients fulfilling ICD C71 diagnosis in our records between 2005 and 2016. We excluded 2 paediatric patients, 36 patients with other histological diagnoses and 29 patients with insufficient follow up information in our registries; therefore, 74 patients were eligible for analysis in the present study (Figure 1). Patients and tumour characteristics are described in Table 1.

In this cohort, 50 patients were males (67.6%) and 24 females (32.4%), with a median age at onset of 58 (range 24–79) years. Eleven (14.9%) have a history of previous systemic malignancy, mainly prostate and breast cancer. Fifty-one has other medical comorbidities. The median time between the onset of symptoms and the first brain scan was 14.5 days, and the median KPS was 80% at the time of diagnosis (82% of patients with KPS ≥ 80).

Twenty-one (28%) patients had comorbidities, with a median CCI score of 2.

### Tumour features

The most common localisation was the frontal (28.4%), temporal (33.8%) and parietal lobes (20.3%). Other less common localisations were the occipital lobe in 5.4% of cases, brain stem (1.4%), basal ganglia (4.1%) and corpus callosum (1.4%). 5.4% were multifocal at the time of diagnosis.

Twenty-three patients were studied with molecular testing, 2 of them showed 1p19q codeletion, 13 MGMT promoter methylation and 1 patient had mutant IDH-1 by PCR/direct sequencing. Only three patients were classified as gliosarcoma (Table 1).

### Treatment

Of a total of 74 patients diagnosed as GBM, 63 underwent surgical resection; 20 (27%) PR and 43 (58.1%) GTR. The remaining 14.9% underwent biopsy only (1 open biopsy and 10 stereotactic biopsy). Eight patients were exclusively treated with palliative care (only symptoms management) due to rapidly progressive disease and/or poor KPS at the diagnosis. Sixty-four (87%) patients received Stupp regimen with a median of 6 adjuvant cycles of TMZ (range 0–19), one patient received chemotherapy only with TMZ after resection and one patient exclusively external beam radiation therapy 30 Gy in 10 fx. Grade 3 and 4 toxicity was mainly haematologic 11 (17.2%) and infectious 5 (7.8%); one patient suffered of liver failure. Two patients showed mild skin allergy related to TMZ.

Fourteen patients (20%) went to reoperation at the time of suspected tumour progression. Of these patients, the median age was 56 years and media KPS at diagnosis was 90%. In two cases, the pathological assessment concluded radionecrosis.

Thirty-six patients went to a second line chemotherapy, 49% received bevacizumab (alone or combined with either irinotecan or lomustine), and 27% were rechallenged with TMZ (two of those under a dose-intense 21/28 schema). The median number of cycles received of bevacizumab was 3 (range 1–10), and 2 (range 1–5) in the case of TMZ.

### Outcomes

The median OS was 13.9 months (standard error (SE) 1.71; 95% confidence interval (CI), 10.56–17.23), 31% were alive at 2 years.

Median PFS was 10 months (SE 1.71; 95% CI, 6.635–13.365) with a 12 months PFS of 36.5% and 18.9% at 24 months of the cohort.

**Figure 2.**
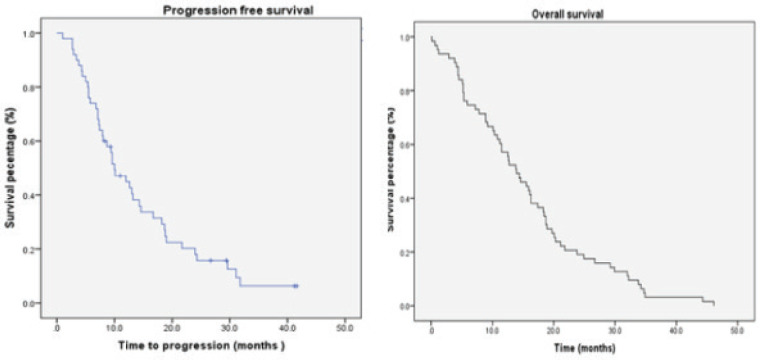
Survival patients treated according to Stupp protocol.

In the subset of patients who received Stupp regimen (*n* = 64), 35.9% alive at 2 years. The median PFS was 10 months (SE 1.8; 95% CI, 6.481–13.519) with a 12 months PFS of 32.8% and 14% at 24 months. The median OS was 16.1 months (SE 1.53; 95% CI, 13.01–19.099). The median SS was 11.8 months (SE 1.61; 95% CI, 8.632–14.968) (Figure 2).

The mean time between surgery and the beginning of radiotherapy was 30 days.

Median survival for patients that had GTR was 16.3 months (95% CI; 2.934–7.466), for patients with subtotal resection, median survival was 12.5 months (95% CI; 10.618–14.382), whereas for those with stereotactic biopsy was 5.2 months (95% CI; 2.934–7.466) (log-rank test: *p* < 0.0001).

Patients with postoperative KPS < 70 had a median OS of 4.4 months (95% CI; 0.242–8.558); on the other hand, patients with postoperative KPS of ≥ 70 had a median OS of 15.9 months (95% CI; 13.454–18.346] (log-rank test: *p* < 0.0001).

Patients who underwent reoperation had a median OS of 32 months (95% CI; 23.819–40.181), which was significantly higher than the median OS of 13.8 months (95% CI; 11.536–16.064) reached by those patients who did not undergo reoperation (log-rank test: *p* < 0.0001). Age at diagnosis <50, 50–60, 60–70 and >70 years, presence of active seizures, presence of comorbidities and CCI did not have statistical significance in the OS (log-rank test: *p* = 0.132; *p* = 0.084, *p* = 0.837, *p* = 0.104, respectively).

Age at diagnosis, presence of comorbidity, CCI, presence/absence of GTR, presence/absence of radiotherapy, concomitant TMZ, postoperative KPS, presence of active seizures and reoperation were assessed at univariable analysis. In univariable analysis, postoperative KPS of less than 60 (*p* < 0.0001; HR 7.3; 95% CI (2.958–18.057)), surgical intervention other than GTR (*p* = 0.001; HR 2.636; 95% CI (1.514–4.590)), absence of radiotherapy (*p* < 0.0001; HR 19.705; 95% CI (6.999–55.474)) and the absence/presence of TMZ (*p* = 0.001; HR 2.867; 95% CI (1.556–5.282)) were significant factors that impacted the OS (Table 2).

In multivariable analysis, the absence of TMZ (*p* = 0.006; HR 4.177; 95% CI (1.519–11.483)) and no reoperation (*p* = 0.011; HR 2.760; 95% CI (1.256–6.065)) significantly increased mortality. The higher postoperative KPS showed a positive trend towards significance (*p* = 0.055; HR 3.117; 95% CI (0.977–9,941)) (Table 3).

## Discussion

This study, at the time, is the most comprehensive clinical series of GBM patients with access to Stupp protocol in Chile. The median OS for the entire cohort was 13.9 months consistent with that of other recent series of developing countries with access to treatment [6–8]. 87% of the patients went to Stupp protocol after surgery, in this group the OS was 16.1 months, similar to the original report [1].

Of the well-known factors associated to the outcome of GBM patients, the postoperative KPS ≥ 70% and GTR (compared to PR or biopsy) showed association to a better survival outcome. The age at diagnosis in our population was younger than the standard GBM population [9] similar to other series of developing countries [6, 7]; however, age at diagnosis was not a significant factor for survival. In this series, we have a limited number of older patients. In our practice, we offer Stupp protocol to elderly of age up to 79 years, decision based on patient’s KPS status, general status and comorbidities.

We also included the CCI used in cancer patients to predict surgical outcome, survival and quality of life [10, 11]. Moraes *et al* [12] reported 48 GBM patients over 80 years and found a direct correlation between CCI ≤ 6 and better OS outcome. In this series, the CCI did not showed relation to OS; it may be explained by the fact that our population is younger with less important comorbidities, with only three patients showing a score of 6 or more.

As a novel issue, we introduced the SS in this cohort as secondary measure of outcome, that means the survival of the patient capable to perform the basic activities of the daily living without help. In the subset of patients who received Stupp protocol, SS followed in 1 month to PFS (10 months PFS and 11 months SS), so the radiologic diagnosis of progression precedes closely the clinical deterioration of our patients.

A marked annual increase of CNS cancer incidence (mainly brain tumours) is seeing in South American countries [2]. This could be explained by the improved access to diagnostic technology or to specific understudied regional raising unknown risk factors. Few studies have examined adult onset GBM in Chile. Lorenzoni *et al* [13] analysed retrospectively 103 patients with high grade glioma treated with radiotherapy (between 1990 and 1999) with an OS of 10 months in GBM patients. In 2011, Quezada *et al* [14] reported a median OS of 6 months in a single public hospital series of 73 patients; none of the patients received either radiotherapy or chemotherapy.

## Conclusion

At the present series, patients treated with Stupp protocol reached a survival similar to the original clinical trial and other single centre reports, showing that disease behaviour is relatively homogeneous. Despite the variability in incidence between regions [2], the OS in this series and in different reports remains similar and depends mostly on access to standard treatment.

## List of abbreviations

GBM, Glioblastoma; TMZ, temozolomide; KPS, Karnofsky performance status; PFS, progression free survival; CI, confidence interval; OS, overall survival; SS, selfcare survival; CNS, central nervous system; CAS, Clínica Alemana Santiago; ICD-10: International classification of diseases 10^th^ revision; WHO, World health organization; MGMT, O^6^-methylguanine-DNA methyl-transferase; IDH: Isocitrate dehydrogenase; REDCap, Research electronic data capture; CCI, Charlson comorbidity index; PR, partial resection; GTR, gross total resection.

## Funding

This research did not receive any specific grant from funding agencies in the public, commercial or not-for-profit sectors.

## Conflict of interest

The authors have no conflicts of interest to declare.

## Figures and Tables

**Table 1.** Patient characteristics and treatment.

	Cohort *N* (%)	Stupp protocol *N* (%)
Number	74	64
Age	58 (24–79)	57 (24–79)
Gender	Male 50 (67.6%) Female 24 (32.4%)	Male 44 (69%) Female 20 (31%)
KPI 100 90 80 70 60 or less	Media: 80% 26 (35%) 7 (9%) 21 (28%) 8 (11%) 9 (12%)	Media: 90% 24 (38%) 6 (9%) 8 (13%) 18 (28%) 3 (5%)
Charlston Comorbidity Index	2 (0–7)	2 (0–7)
Clinical Presentation Asymptomatic Headache Seizure Cognitive impairment Focal sign	2 30 22 28 39	2 28 22 22 30
Surgery Gross total resection Partial Resection Biopsy only	43 (58.9%) 19 (26%) 11 (15.1%)	40 (63%) 18 (28%) 6 (9%)
Therapy 1rst line Palliative Radiotherapy Chemotherapy Stupp Regimen	8 (10.8%) 1 1 64	0 0 0 64 (100%)
Molecular Markers Met MGMT IDH 1 IDH 2 1p/19q	13/20 (65%) 1/21 (4.8%) 0/21 2/6	12/19 (63%) 1/20 (5%) 0/20 2/5

**Table 2.** Cox univariable regression for overall mortality.

	Standard error	Hazard ratio	95% confidence interval	*p*-value
No Temozolamide	0.312	2.867	1.556–5.282	0.001
KPI < 60	0.461	7.308	2.958–18.507	<0.0001
No GTR	0.283	2.636	1.514–4.590	0.001
No reoperation	0.365	3.581	1.752–7.321	<0.0001
No radiotherapy	0.528	19.705	6.999–55.474	<0.0001

KPI: Karnofsky performance index GTR: Gross total resection.

**Table 3.** Log-rank test multivariable analysis for overall mortality.

	Standard error	Hazard ratio (HR)	95% confidence interval	Significance
No Temozolomide	0.516	4.177	1.519–11.483	0.006^a^
KPI < 60	0.592	3.117	0.977–9.941	0.055
No GTR	0.317	1.311	0.704–2.441	0.393
No reoperation	0.402	2.76	1.256–6.065	0.011^a^
No radiotherapy	0.768	2.169	0.481–9.768	0.313

a KPI: Karnofsky performance index; GTR: Gross total resection.
